# Waiting in crowded places: influence of number of pedestrians, waiting time and obstacles

**DOI:** 10.1098/rsif.2023.0193

**Published:** 2023-09-13

**Authors:** Mira Küpper, Armin Seyfried

**Affiliations:** ^1^ School of Architecture and Civil Engineering, University of Wuppertal, Wuppertal, Germany; ^2^ Forschungszentrum Jülich, Institute for Advanced Simulation, Jülich, Germany

**Keywords:** experiment, pedestrian, waiting, train station

## Abstract

At crowded places, like railway platforms at rush hour, the spatial distribution of waiting pedestrians has a significant influence on performance and level of comfort. However, the choice of waiting places and the resulting spatial distribution of the crowd have rarely been studied. This study investigates the effects of obstacles, number of passengers and waiting time on the distribution of waiting passengers. Laboratory experiments were performed using a mock-up platform with three set-ups: without obstacles, with a narrow and a wide obstacle. Density profiles determine preferred waiting places. While the space usage by waiting passengers is inhomogeneous, the distances between the individuals show surprisingly small variations, regardless of obstacles and number of passengers. This suggests a robust collective optimization of the crowd when searching for waiting positions. In doing so, and without necessity, the participants chose distances to each other extending into the personal zone specified in classical personal-space-concepts. These results indicate necessary refinements of the concept by considering context and collective behaviour. The findings are transformed into floor-fields modelling the space usage by a superposition of attractive or repulsive areas which consider optimization of distances and comfort. This model does not only reproduce the waiting places at platforms but can be adapted for other use cases.

## 1. Introduction

The research field of pedestrian dynamics studies the movement and behaviour of pedestrians. For an overview the reader is referred to the biannual conference series [1,2], the reviews on the topic, e.g. [3–5] and the glossary on human crowd dynamic research [6]. While most studies are focused on pedestrians walking through, e.g. corridors, bottlenecks or in evacuation scenarios, situations in which pedestrians are waiting or standing still for a while were subject of fewer investigations. However, standing or waiting pedestrians are present in many daily situations, such as at public transportation facilities where passengers wait at railway platforms or inside trains.

Especially under the assumption of an expected increase of passenger numbers in public transport facilities in central Europe in the near future, the waiting behaviour and distribution of boarding passengers is of interest as standing pedestrians narrow the available space and influence the overall performance of the station. At train station platforms, the boarding passengers usually spend some time waiting and thereby show a different space usage than moving pedestrians. The distribution of passengers along the platform is of importance for this matter as uneven distributions of passengers lead to non-uniform uses of the train doors in the boarding process, which can increase the train’s dwell times and influence the performance of the station [7–9].

Previous studies on passengers' waiting behaviour at railway platforms found that the passenger distribution is not uniform along the platform but influenced by the location of the entry ways [10–14] and stopping positions of the trains [15]. Passengers tend to cluster around the entrances (cf. [11,16–18]) or infrastructure elements (like seating arrangements or vending machines [19–21]), beginning with the ones closest to the entrance. Waiting places close to obstacles were found to be preferred as those offer the possibility to lean against them cf. [22,23]. In regions close to obstacles passengers accept higher densities while waiting [22,24]. At a two-sided platform usually only regions at the platform side of the expected train arrival are occupied [24]. Spaces close to the platform’s edges are avoided [25].

Moreover, different types of passengers exhibit different preferences for their waiting locations. Passengers travelling in social groups often choose waiting places that are wide enough for the whole group to fit and ensure the groups communication. This is often the case in areas directly in front of the entrances, leading to possible bottlenecks and congestion [26]. Passengers with short head time to the expected train arrival stand in region close to the entrances, while passengers with longer waiting times prefer undisturbed spaces and even walk to farther platform areas or use the rearward sides of stairways [26]. Commuters often develop individual strategies in order to minimize the distance to the exits at their destination [27].

The studies introduced in the previous paragraphs highlight findings obtained in field observations on passengers using platforms at railway or underground stations. If this situation is abstracted, it can be described as pedestrians entering a space through an entrance, waiting for a certain amount of time and then leaving the space through an exit. This situation is comparable with pedestrian’s inflow into confined spaces and happens in daily life, e.g. in elevators, waiting rooms or terminals at airports. Laboratory experiments, which can reduce the complexity of influencing factors, aiming at investigating pedestrian’s waiting behaviour and distribution inside confined spaces were performed and analysed by [28–33]. In contrast to situations in which the walking direction is predefined (and e.g. given by the experiment’s instructions or set by the goal that should be reached), in inflow situations pedestrians can freely choose their walking direction and waiting place. Upon entering a room, a pedestrian needs to decide on a waiting place taking into account the current situation which is, among some potential personal preferences, composed of the current position of other pedestrians, the expected filling of the room (by further entering persons) and the expected exit from the room.

Experimental studies on inflow processes into a room were previously performed with two different set-ups: (a) for square rooms (approx. 4 × 4 m) with one door that serves as both entry and exit (like e.g. an elevator) [28,29] and (b) for a rectangular room (approx. 2 × 10 m) with separate entry and exit doors [30–33].

— In set-up (a), it is reported that the filling of the room starts at the boundaries beginning with the wall on the opposite side of the door. Subsequent persons fill the room forming an arch around the boundaries. The reasons proposed for those boundary preferences are the option to lean against the walls and less repulsion and contact to other pedestrians [29]. With increasing number of pedestrians inside the room the middle parts are used and the final distribution of participants in the room is uniform with only slight variations in Voronoi cell sizes [28]. After reaching their stopping positions pedestrians turn towards the exit and are thus facing subsequently entering persons. It was observed that pedestrians mostly remain standing at the position in which they first stopped inside the room and only small fluctuations occur [28]. The authors of [28] identified four main factors which influence the decision-making process in inflow situations. These were named: flow avoidance, distance cost, angle cost and boundary preferences.

While these findings refer to non-competitive situations, [29] showed that the distribution of participants changed significantly when including a first-out-award. In this scenario, pedestrians gather close to the exit and the average distance to the nearest neighbour decreases. The participants entering first can minimize their distance to the door in order to obtain an advantageous exit position, which leads to interference with subsequently entering pedestrians.

— In set-up (b), the rectangular room has a separate exit door located near the middle of the longer wall. Pedestrians entering first chose a position close to the exit doors [32]. In contrast to the observations in set-up (a) no boundary effect was reported in the set-up with a separate exit door. However, in the images shown in [31–33], the barriers used to mark the experimental set-up in scenario (b) appear to be soft (traffic cones and ropes) and therefore would probably not offer the same function as solid walls which can provide comfort through the possibility to lean against them and safety as those will decrease the number of neighbouring pedestrians, especially of those standing directly behind. The final distribution of participants in the room was not uniform, as participants gathered in the middle of the room between the entry and exit doors and hence higher densities are reported in those regions [30,32,33]. The far side of the room was not used [31]. This results in a spatial distribution that is not even throughout in the whole available space.

The first proposed explanation for these findings is that the distribution of waiting passengers is related to the personal space as participants perform a trade-off between the desire to optimize their distance to the exit and to preserve their privacy [32,34]. The preservation of privacy is described by psychologists in the concept of personal space; e.g. [35,36] characterize this as the area in which the entering of other persons causes discomfort. The area surrounding an individual is divided into different zones: the intimate (*d* < 0.45 m), personal (0.45 < *d* < 1.2 m), social (1.2 < *d* < 3.6 m) and public (*d* > 7.6 m) zones [35,36]. The personal distance (0.45 < *d* < 1.2 m) is in this context often described as the distance that individuals try to maintain between themselves and others [36] and is used in many approaches to model the movement of pedestrians and crowds. The distance zones in these concepts were determined between persons of varying degrees of familiarity (e.g. friends or strangers) standing face to face in different settings (e.g. office, street corner) [35]. Goffman investigated the relation of people in public spaces [37] and describes the process as follows: pedestrians entering first can freely choose their waiting location but afterwards each newly entering person causes the others to shift position and relocate sequentially. With an increasing number of pedestrians inside a space, the amount of free space decreases and pedestrians are distributing more uniformly, eventually reaching a state of equal distances. Those equal distances are achieved by self-organization processes, which were previously described as a weighting up between the desire to allocate the space equally and to maximize the distance to others. However, recent studies found that increasing densities and the associated violations of personal space do not simply increase the discomfort perceived in those situations. In low densities, where the available space is higher, the discomfort caused by invasion of personal space is higher [38].

Comparing the studies on waiting passengers at railway platforms and in inflow situations, the behaviour of the pedestrians shows many similarities. In both cases short distances (to the entrances or destinations) are preferred, and waiting places are often located close to the boundaries. However, at railway platforms the boundary preferences correspond to obstacles or other infrastructural elements rather than the platform’s edges. In contrast to boundaries of spaces enclosed by walls, the platform edges are hazard zones and therefore avoided by passengers. As far as distances to destinations are concerned, usually in enclosed spaces the exact location of the door is visible and known. On platforms, the exact location of the targets, in this case the train doors, may be unknown due to the unpredictable stopping points of the trains.

Passengers’ waiting behaviour at railway platforms was until now studied using field observations. As reviewed in the section above a large variety of factors (e.g. location of entrances, infrastructural elements, stopping position of trains etc.) was found to have an influence on passengers' choice of waiting positions. However, in observations of real-life situations it is not possible to distinguish the influences of different factors separately. Therefore, this study investigates the influence of obstacles, waiting time and number of passengers on the distribution at railway platforms by performing laboratory experiments. These factors were chosen as they were expected to have a relevant influence on the distribution of passengers and were technically possible to investigate within the framework of a given set of laboratory experiments (for details see Methods section and [39]).

After an introduction of the experiment procedure and the data preparation, trajectories and density profiles are presented in order to show passengers' waiting places and distribution along the platform. The distances kept by waiting participants and the perception of the experimental runs are shown. Based on the results obtained from this analysis, a floor-field model for waiting places is developed, which can also be adapted for other scenarios than train station platforms.

## 2. Results

### 2.1. Experiments and data collection

**Figure 1.**
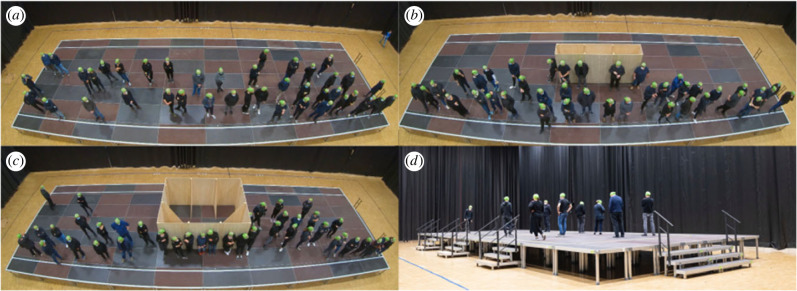
Experimental set-up and participants’ positions at the end of the waiting time shortly before the time of announcing the trains’ arrival. (*a*) Platform without obstacle, (*b*) with narrow obstacle, (*c*) with wide obstacle. (*d*) Side-view of the experimental set-up. At the right-hand side, the entrance is located, at the left side, the movable stairs that act as train doors are visible.

The experiments were performed by using a mock-up train station platform (for details see Methods section). The platform was either equipped with no obstacle, a narrow or a wide obstacle as seen in figure 1. Each set-up was tested with 40 and 100 participants. Participants entered the platform through stairs at the right-hand side of the platform (figure 1*d*), and waited for a train that was to arrive at the platforms lower side. In runs with 40 participants the waiting time, which started after the last participant of the run had entered the platform, was either 2 or 4 min; in runs with a higher number of participants the waiting time was 2 min. After the waiting time was completed, the train arrival was announced, movable stairs, which acted as train doors (see figure 1*d*, left-hand side), were positioned and the participants left the experimental side. Each scenario was repeated three times with different participants. After each run, participants were asked to use a mood button terminal to express how they felt during the experiment. After selected runs questionnaires were distributed; see table 1 for an overview. Participants’ head trajectories were automatically extracted following [40] using the software PeTrack [41], which is achieved by recognizing and tracking of green caps worn by the participants. For details on procedure and data collection, see Methods section.

**Table 1.** Overview of the experiment runs.

set-up	no. participants	waiting time (min)	questionnaire
no obstacle	40	2	no
no obstacle	40	4	no
no obstacle	100	2	yes
narrow obstacle	40	2	yes
narrow obstacle	40	4	yes
narrow obstacle	100	2	no
wide obstacle	40	2	yes
wide obstacle	40	4	yes
wide obstacle	100	2	no

### 2.2. Trajectories

The exemplary images from an overview camera seen in figure 1*a*–*c* show a time frame at the end of the 2 min waiting time for runs with 40 participants and the three different platform set-ups. Observable in all set-ups is that the participants do not distribute evenly along the platform, but cluster at the lower side. Also their viewing direction is turned towards that side, which is caused by their awareness of the side of train arrival.

**Figure 2.**
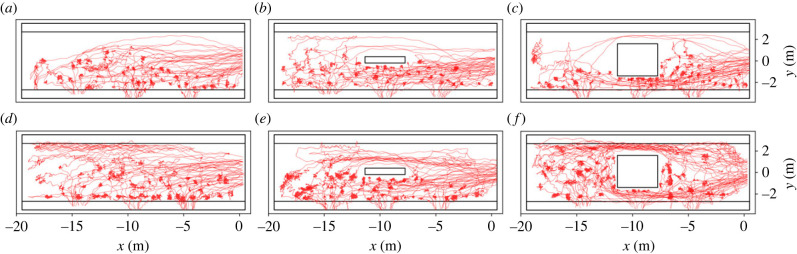
Trajectories for exemplary runs with 40 participants for the three different set-ups, a platform without obstacle, a narrow or a wide obstacle. Waiting time was either 2 min (*a*–*c*) or 4 min (*d*–*f*).

Figure 2 shows the trajectories of exemplary runs with 40 participants for the different set-ups; in the upper panel (*a*–*c*) the waiting time of 2 min, in the lower panel (*d*–*f*) runs with 4 min waiting time. In runs with 40 participants and 2 min waiting time, the upper side of the platform is only used for walking to the desired waiting spots. Waiting places can be identified in the trajectories as ‘knots’, since standing pedestrians still show slight head movements. Those waiting places are mainly located at the side of the lower platform, where the train is expected to arrive. This phenomenon is more pronounced in runs with obstacles than in runs without obstacles. With an increased waiting time of 4 min (figure 2*d*–*f*) the area covered by trajectories increases, as some participants start to walk around instead of waiting at a fixed position. The moving participants can be identified by the wave-like trajectories, which are mainly located at the side of the platform which is free from standing participants. In all runs with obstacles, independent from waiting time, participants waiting at a fixed position can be observed to lean against the sides of the obstacles. However, the obstacles’ sides facing towards the side of train arrival are used more frequently than the sides facing towards the smaller sides of the platform.

It should be noted that in all runs the safety line is only rarely crossed and no participant waited in the area between the safety line and the platform’s edge, even though participants were not told to pay attention to these lines indicating danger zones.

### 2.3. Density profiles

The density was calculated using the Voronoi method following [42]. For each pedestrian *i* a Voronoi cell is defined as the area that is closer to the given pedestrian than to all others. The Voronoi cells are cut at the edges of the platform, therefore no open cells are existing at the boundaries. The density *ρ* corresponds to the inverse of the area of the Voronoi cell *A*_*i*_ and is calculated for each frame

2.1 $$\rho_{xy}=\frac{1}{A_{i}}\;\mathrm{if}\;(x,y)\in A_{i}.$$

Voronoi density *ρ*_*V*_ of a measurement area *A* is then calculated as

2.2 $$\rho_{V}=\frac{\iint \rho_{xy}\;dx\;dy}{A}.$$

To perform a spatial analysis of the data, as introduced in [43], the measurement area is parcelled into tiles with the size of 0.2 × 0.2 m. For each frame within the waiting time, the Voronoi densities were calculated and integrated over time for each tile. The calculation of Voronoi densities and profiles was carried out using the Python library PedPy [44] and the software JPSreport [45]. Density profiles were calculated for the waiting time in each of the three repetitions of the experimental runs and averaged over the number of frames of the waiting time.

**Figure 3.**
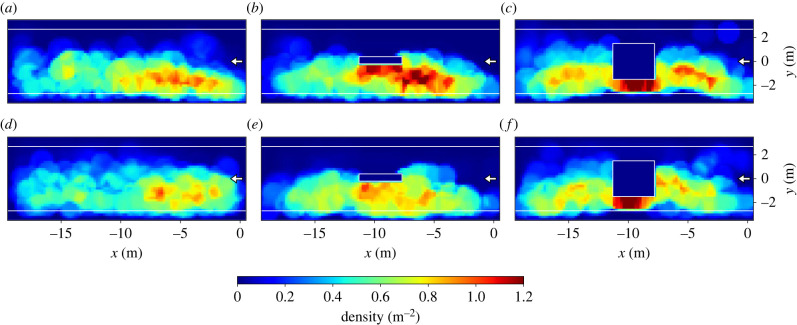
Density profiles for *N* = 40 participants. (*a*–*c*) 2 min waiting time; (*d*–*f*) 4 min waiting time. The location of the entrance is marked with a white arrow.

In experiments with 40 participants (see figure 3), the side of the expected train arrival is visible for all configurations as participants mainly distribute themselves along the lower platform side. In the case of 2 min waiting time (figure 3*a*–*c*), the areas of highest densities for a platform without obstacle are shifted towards the entrance at the right. With obstacles the density distribution becomes more even in *x*-direction. In these runs, the highest densities are found close to the obstacles. Compared with the profiles for runs without obstacle, more space farther away from the entrance is covered and concurrently the side facing towards the entrance is used more often.

**Figure 4.**
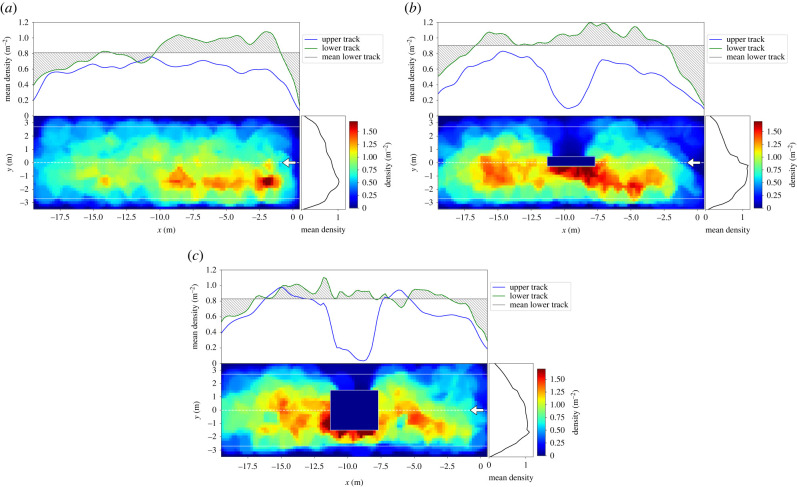
Density profiles and mean density in *x*- and *y*-direction for runs with 100 participants. Mean densities in *x*-direction (upper panel) are separated into the different track sides. For the lower track (side of train arrival), the mean density is indicated as grey line, the density deviation from the mean is highlighted as grey area for (*a*) the platform without obstacle, (*b*) with narrow obstacle and (*c*) with wide obstacle. The location of the entrance is marked with a white arrow. (*a*) no obstacle. (*b*) narrow obstacle. (*c*) wide obstacle.

The sides of the obstacles facing towards the lower track exhibit the highest densities with up to 1.3 m^−2^. In the set-up with the wide obstacle, those areas extend towards both sides of the obstacle along the platform. It is pointed out that the differences are clearly discernible but small ranging between approximately 0.6 and 1.3 m^−2^. Comparing the runs with shorter (2 min) and longer (4 min) waiting times, it seems that for the latter the density becomes more homogeneous and more space is covered, cf. figure 3*d*,*e*. With an increased waiting time of 4 min some participants start to walk slowly instead of waiting at a fix location, see figure 2*d*,*e*. Consequently, and regardless of the presence of obstacles, the densities decrease as the participants cover more of the available space. To analyse the inhomogeneity of the density, the density profiles for runs with 100 participants as well as the density distributions along the *x*- and *y*-axis are shown in figure 4. Density distributions are calculated as a sum of densities for the tiles in the corresponding direction and averaged over the number of accessible tiles. Since the knowledge of the side of train arrival structures the distribution of densities along the platform, the density distributions (upper plots) are separated into the lower track (green line) and the upper track (blue line). The mean density at the lower platform side is illustrated as grey horizontal line and the deviation of the density distribution of the lower track from the mean density as grey area. This means that grey areas above the line of mean density indicate that a platform region is showing a density above average.

Comparable to the runs with 40 participants the highest densities for the platform without obstacle are located at the side of the entrance on the lower track, causing the density distribution to show a positive deviation from the mean density. The distribution at the upper track is more even.

In the runs with obstacles (figure 4*b*,*c*), the sides of the obstacles were preferred waiting locations and therefore shift the density distribution towards the lower side of the track. This is most distinct for the narrow obstacle (figure 4*b*) right side). In the case of the wide obstacle in runs with 40 participants mainly the sides facing towards the lower track were used for waiting. With an increasing number of participants, the sides facing towards the smaller sides of the platform (left and right in figure 4*c*) became more attractive as waiting places. This leads to a more even distribution along the platform. However, the rearward sides of the obstacles (which are facing towards the opposite track) are unattractive and the density distributions exhibit a distinct drop in the curves of the upper track (blue lines) at the location of the obstacles. Those areas are unattractive because there is no direct line of sight to the next expected action, which was the ‘boarding’ of the train at the lower track. Hence, narrow obstacles structure the distribution of densities towards the lower platform side as those visually separate the track sides, while in the case of wide obstacles more space along the platform (in *x*-direction) is covered. This causes obstacles to have a two-sided effect: the side facing towards the lower side of the platform (train arrival) is attractive while the opposite side acts as repulsive. Despite the density variations along the platform, a once chosen waiting place is only seldom changed even if the density in other regions is lower.

### 2.4. Distribution of distances

Concerning the distribution of pedestrians, two different definitions on uniform distributions can be given: pedestrians can be evenly distributed in space, which would be characterized by a coverage of the whole available space and concurrently by equal sizes of Voronoi cells. However, the results presented in the previous sections show that in the case of a railway platform the awareness of the train arrival causes the participants to congregate at one side of the platform and therefore the distribution is non-uniform in space. Nevertheless, the distribution of participants inside the crowd can still be uniform, which is identified by equal interpersonal distances without completely covering up the available space.

**Figure 5.**
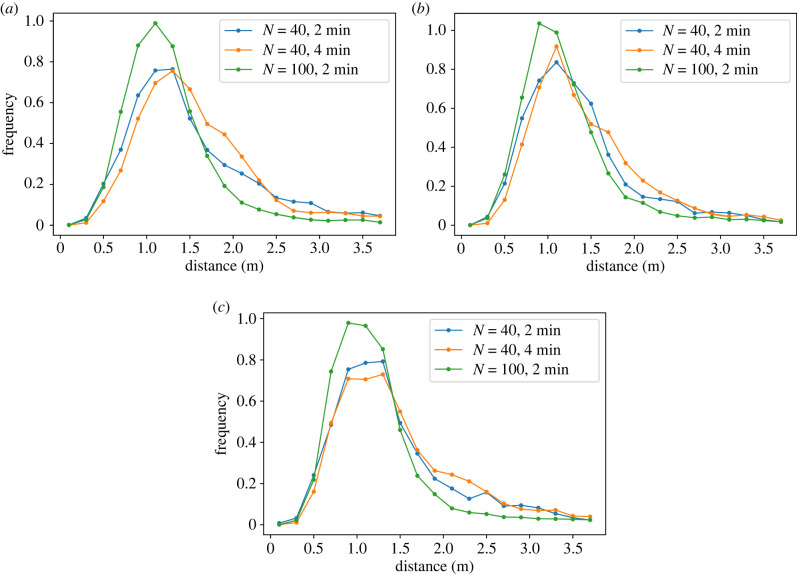
Histograms of distances between participants for (*a*) the platform without obstacle, (*b*) with narrow obstacle, (*c*) with wide obstacle. Distances were calculated with Delaunay triangulation during the waiting time.

The distances between neighbouring participants, identified using Delaunay triangulation, were calculated for each frame within the waiting time and visualized as histograms; see figure 5.

For each set-up (no, narrow or wide obstacle), the distances were determined for runs with 40 participants and 2 min waiting time (blue), 4 min waiting time (orange) and for 100 participants (green). The distribution of distances differs in runs with 40 participants for the two waiting times. Tukey tests reveal that this difference is significant (*p* < 0.001). For all set-ups, the difference becomes visually apparent in the interval of distances between 1.6 and 2.5 m. (figure 5*a*–*c*). For 4 min waiting time, these distances are more frequent, as those correspond to pedestrians that walk slowly instead of waiting at a fix position.

The distributions of distances for runs with 100 participants differ significantly from the runs with a lower number of participants (*p* < 0.001). With an increasing number of persons the histograms become more symmetric around a mean value of about 1.0–1.2 m with only small fluctuations (s.d. ≈0.25 m). The skewness to the right, corresponding to larger interpersonal distances, becomes smaller with increasing passenger numbers. This can be quantified as for 40 participants and 2 min waiting time a portion of about 30% of the distances are greater than a threshold of 1.6 m, while with an increasing passenger number this portion decreases to 15%.

Assuming theoretically that the persons would be evenly spaced out like aligned on a grid over the whole available area, the interpersonal distances would be 1.9 m for runs with 40 participants and 1.18 m for 100 participants. However, all histograms of distances show distributions around 1.0–1.2 m. Despite the larger available space per person the participants do not use this space; see the density profiles in §2.3. It is unclear whether the interpersonal distances will also be in this range if the number of participants is increased even further or decreased to fewer than 40 participants. As previously discussed in §2.3, the density ranges mainly between 0.6 and 1.2 m^−2^ and therefore corresponds to a range of distance of 1 to 1.4 m, respectively.

The above findings and discussions imply that in these experiments the interpersonal distances are uniform, mostly independent of the global density, number of passengers and the complexity of the waiting areas. Even in situations in which wide obstacles or other pedestrians block the view, waiting pedestrians achieve uniform interpersonal distances. The preference of pedestrians to maintain equal distances between each other is already described in [37]: passengers in public environments are expected to keep a distance from one another and thus cover the space equally. Following the zones of interpersonal distance introduced by [36], the distances observed in the experiments are without any need inside the ‘personal zone’ 0.45 m < *d* < 1.2 m and hence smaller than expected for a public environment with a social distance of 1.2 m < *d* < 3.6 m. This is an indicator for a superposing effect which seems to surpass the desire to maintain the personal distance to other pedestrians. In the experiments, the awareness of the next action seems to cause the reduction of the distance to exit, and the concept of maintaining the personal space does not appear to be the predominant factor in these situations. However, the thresholds determining the extent of the personal space in [36] were set for situations between a pair of pedestrians facing each other. In the case of waiting passengers at a train station, the passengers are aligned behind each other, usually facing towards the side of the train arrival. It is therefore questionable if these distances can be adopted for waiting situations or whether a refinement of the concept of personal space is necessary. As these concepts are widely used in the modelling of pedestrian movements, this should be further investigated.

### 2.5. Rating of experiments: questionnaires and mood button terminals

Runs with obstacles for 40 participants and runs with 100 participants without obstacles (cf. table 1) ended with the participants being asked to answer questionnaires. Participants were asked to indicate their perception of the density and available space during the experiment. Despite the varying number of participants, the rating of density and available space is always very positive and does not exhibit suitable variances. This might indicate that the density differences achieved in the experiments were not high enough to cause discomfort among the participants.

**Figure 6.**
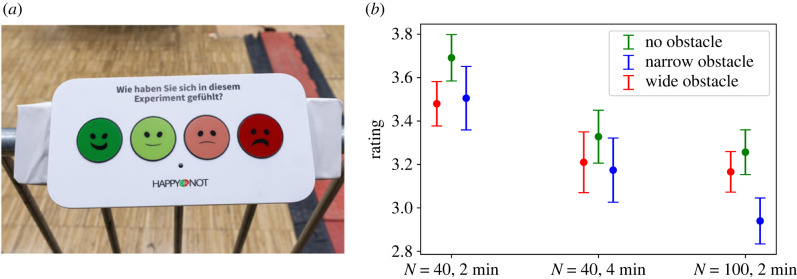
(*a*) Mood button terminal: participants were asked to rate the latest experiment using the smiley buttons. The question on the terminal was (translated from German): ‘How did you feel during this experiment?’ (*b*) Rating of perception of experiments: 4 = `very happy′, 3 = `happy′, 2 = `unhappy′, 1 = `very unhappy′ (error bars: 95% confidence interval)

While the questionnaires specifically asked for the participants’ perception of density and space, the mood button terminals (figure 6*a*) asked about the overall perception of the experimental run. This could include, for example, the perception of e.g. waiting time or boredom of a single experimental run or the tiredness due to the running time of the sets of experimental runs.

In order to analyse the participants’ overall assessment of the runs, see figure 6*b*, the experiments were divided into three groups: (a) runs with 40 participants and 2 min waiting time, (b) 40 participants and 4 min waiting time and (c) 100 participants and 2 min waiting time. Each of these groups contains runs of the three different set-ups (no, narrow and wide obstacle). The effects of set-up and waiting time (between groups a and b) and set-up and number of participants (groups a and c) were analysed using ANOVA. To facilitate the analysis, numbers were assigned to the smiley-buttons (cf.figure 6*a*) starting with ‘1 = very unhappy’ and ranging to ‘4 = very happy’. For runs with 40 participants the waiting time has a significant effect on the rating (*F*_1694_ = 13.53, *p* = 0.0003), while the set-up has no significant effect. This indicates that an increasing waiting time leads to less positive ratings. However, mean values higher than 3.0 are still assigned to the ‘happy’ side of the scale.

In runs with 2 min waiting time (groups a and c), both number of participants (F_1,1030_ = 50.98, *p* = 0.000) and set-up (F_2,694_ = 6.92, *p* = 0.0001) have a significant effect on the rating. Their interaction effect is also significant (F_2,1030_ = 4.22, *p* = 0.0001). Therefore, an increase of the number of participants decreases the rating. A *post hoc* test revealed significant pairwise differences between the set-ups with the narrow obstacle to both other set-ups. In runs with 100 participants the set-up with the narrow obstacle (*M* = 2.94, s.d. = 0.88) was rated significantly poorer than the runs without obstacles (*M* = 3.26, s.d. = 0.73, *p* = 0.001) and with wide obstacle (*M* = 3.17, s.d. = 0.74, *p* = 0.004). A mean value smaller than 3.0 for the narrow obstacle places the participants’ assessment of these runs on edge towards the ‘unhappy’ side of the scale.

Hence, the mood button terminals reveal that both an increasing waiting time and an increasing number of participants (here by more than a factor of two) have a comparable negative influence on the participants’ overall perception of the experiments. The three set-ups (no, narrow and wide obstacle) have no significant effect on the rating in runs with 40 participants. However, the narrow obstacle shows significantly lower ratings in runs with 100 participants. Possible reason for this is the structuring effect of the wall-like obstacle which causes the participants to mainly wait at the lower side of platform. It should, however, be noted that the experiments with 100 participants and the narrow obstacle were performed directly before participant’s lunch break and therefore a tiring effect cannot be ruled out.

### 2.6. Floor-field model for waiting

**Figure 7.**
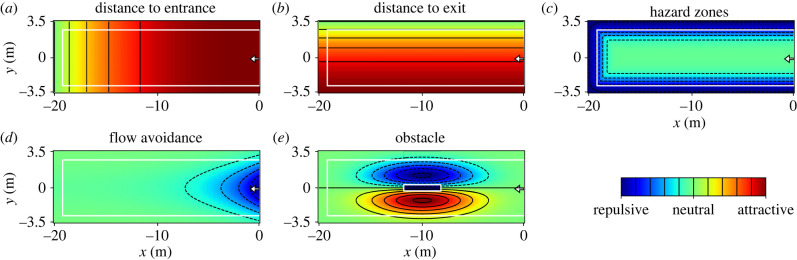
Schematic illustration of influencing factors: (*a*) distance to entrance (*b*) distance to exit (*c*) repulsion of hazard zones (*d*) flow avoidance (*e*) effect of obstacle. Colours indicate the attractiveness as waiting place with blue marking areas as repulsive and red marking areas as attractive. The location of the entrance is marked with a white arrow.

Based on the results shown in the previous sections, pedestrians are expected to choose their waiting positions based on a trade-off between different factors, which can either act as attractive or repulsive. As an extension of the approaches introduced in [28,46], the following factors (as illustrated in figure 7) were identified to influence waiting pedestrians at a platform. These factors are transformed into floor fields which were calculated by equations and functions designed to represent the results obtained in the experiments. The equations of the floor fields were estimated qualitatively as educated guesses and do not claim general validity. The model is a first approach to test qualitatively if it is possible to describe the attractiveness of waiting places using a superposition of floor fields. Due to the necessary simplifications that were used in the experiments, the model in its current form does not claim to be complete with respect to real platforms, since several factors such as e.g. lighting, seating arrangements or information boards are not included. These would influence certain pedestrians individually, resulting in the need to adapt the floor fields for different passengers. As the conflict between parsimony and accuracy becomes larger with more complex models, the floor fields were not validated or quantified by simulations but should act as first attempt of a floor model for waiting places.

**Figure 8.**
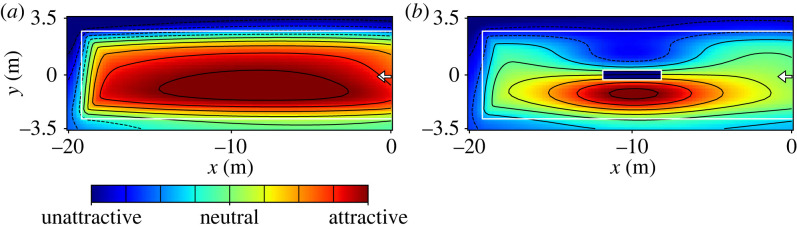
Floor field of attractiveness of waiting positions obtained as superposition of effects from figure 7: (*a*) platform without obstacle (*b*) platform with narrow obstacle. The location of the entrance is marked with a white arrow.

(a) Distance to entrance (figure 7*a*): as already shown in previous studies (cf. [10–14,16–18]) and also observable in the laboratory experiments, passengers prefer staying close to the entrance and do not walk to the far side of the platform. This behaviour is also reported by [28] for inflow situations into confined spaces and leads to an increased attractiveness of areas in the vicinity of the entrance. The equation used to calculate the distance cost was chosen to be maximal at *x* = 0 (position of the entrance) and decreasing as *x*^3^ towards the end of the platform. The power of three was chosen in order to describe a decay in attractiveness of places at the far platform side, while still rating regions in the middle of the platform as attractive. Following the principle of Occam’s razor, the most simple equation was used to describe this behaviour.
  2.3 $$D(x)={\left(x+x_{\min}\right)}^{3},$$
  where *x*_min_ is the position of the end of the platform. The *y*-coordinate of the entrance was left out for simplification as the entrance is almost as wide as the set-up.
(b) Distance to exit (figure 7*b*): in the context of railway platforms, the location of the exit is known to the passengers as the side of the next intended action (usually the boarding of a train). Therefore, waiting places on the corresponding side are more attractive than on the opposite side of the platform. The attractiveness based on the side of train arrival is calculated as
  2.4 $$T(y)={\left(1+\exp(-a\cdot y)\right)}^{-1},$$
  with *a* being a positive constant. Waiting places at the side of train arrival are consequently assessed equally, while the attractiveness decreases exponentially from the middle towards the opposite track. As the side of train arrival but not the exact position of the doors is known, the *x*-coordinates of the doors are not part of the equation. In the case where a platform with installations such as platform edge doors or other visible indications for the exact location of train doors is modelled, this field will probably need to be modified accordingly.
  The factors (a) and (b) are both optimizations of distances which are generally applicable in different set-ups like at train platforms, elevators or rooms/corridors. To a certain extent in all these scenarios, the distances to the entry and exit will influence the distribution. The distance to the exit is expected to have a greater influence than the distance to the entrance, as pedestrians usually know that they will leave at a certain time and therefore the distance to the exit must to be covered anyway. Additionally, a waiting place close to the expected location of the train’s doors might improve chances to get a seat on the train. Besides the optimization of distances also the comfort is an important factor. Here, the context of the situation gains in significance.
(c) Repulsion of hazard zones (figure 7*c*): in contrast to waiting in enclosed spaces, where the boundaries are preferred places [28,29], the platform’s edges have a repulsive effect. Due to the risk of falling, passengers in the experiments kept a distance to the edges and did not wait in the area between the white safety line and the platform’s edge, even though they were not instructed to respect the safety line. The floor field of the edges depends on the distance to the safety line,
  2.5 $$E={\left(1+\exp(-b\cdot B_{ij})\right)}^{-1},$$
  where *B* is a field containing the distance to the safety line and *b* a positive constant. Hence, the repulsion effect of the platform’s edge decreases exponentially from the safety line towards the inner part of the platform and has no effect in the middle of the platform.
(d) Flow avoidance (figure 7*d*): as passengers prefer places where they do not get in the way of others and are not perceived as an obstacle, the area directly in front of the entrance also has an repulsive effect. In real-life field data, this effect will be more pronounced than in the laboratory experiments discussed here, as usually passengers will arrive continuously, while in the experiments no new participants entered the platform during the waiting time. As introduced in [28], the flow avoidance can be described as
  2.6 $$F(x,y)=(-c\cdot \exp\left(\frac{-{\left(x-x_{0}\right)}^{2}}{d^{2}}-\frac{{\left(y-y_{0}\right)}^{2}}{e^{2}}\right),$$
  with *c*, *d* and *e* being positive constants and *x*_0_ and *y*_0_ the location of the entrance.
(e) Stationary obstacles (figure 7*e*): obstacles have a two-sided effect on waiting passengers. The side facing the expected boarding direction is an attractive waiting place, while, due to the restricted line of sight, the opposite side acts as repulsive. Hence, the resulting floor field depends on the location of the obstacle whose corner coordinates are given as *x*_1_, *x*_2_, *y*_1_ and *y*_2_.
  2.7 $$O(x,y)=f\cdot \left(y-\frac{y_{1}-y_{2}}{2}\right)\cdot \exp\left(-\frac{\left(x-x_{1})\cdot (x-x_{2}\right)}{g^{2}}-\frac{\left(y-y_{1})\cdot (y-y_{2}\right)}{h^{2}}\right),$$
  with *f*, *g* and *h* being positive constants. The function is designed to generate negative (repulsive) values for the area behind the obstacle and positive (attractive) values directly in front of the obstacle. With greater distance to the obstacle its influence decreases to neutral values.
  Using a superposition of these factors, a rough estimate of attractiveness of waiting places at railway platforms can be generated. Therefore, the floor fields shown in figure 7 were summed up as *A* = *w*_1_ · *D* + *w*_2_ · *T* + *w*_3_ · *E* + *w*_4_ · *F* + *w*_5_ · *O* using different weights *w*_*i*_ so that different strengths can be assigned to the factors depending on the context.
  The resulting superposition is illustrated in figure 8, where (*a*) shows the platform without obstacles and (*b*) the platform with a narrow obstacle. The weights were set as *w*_1_ = 1, *w*_2_ = 2, *w*_3_ = 3, *w*_4_ = 1 and *w*_5_ = 0 for figure 8*a*) and *w*_5_ = 3 for figure 8*b*). Regions are coloured based on their attractiveness, with red colours marking attractive and blue colours unattractive waiting places. Even though the weights and floor fields were only determined as educated guesses, the resulting patterns are comparable to the results of the experiments. The weights chosen in this example do not claim general validity, but indicate that the distance to the exit and the hazard zones are likely to play a stronger role.
  The suggested floor fields can be used to get a qualitative impression of the final waiting positions, but do not claim to reproduce the dynamics and relocation during the filling processes. In a real environment, the individual factors discussed in the beginning of this section should be added. The floor field can act as a basis for simulation studies to determine pedestrians’ positioning goals. In order to model the distribution of pedestrians along these fields, additionally the interactions between the pedestrians, such as collision avoidance or keeping personal distances, need to be considered. With the work presented here, an example of a floor field of the pedestrians’ desired waiting places is given.

## 3. Discussion and conclusion

This study investigated the influence of obstacles, number of passengers and waiting time on the distribution of waiting pedestrians. As the participants were informed about the side of train arrival, their attention was drawn towards this side and their viewing direction was aligned towards the expected train arrival.

Obstacles were identified to structure the distribution of waiting passengers as their side facing the side of train arrival has a pulling effect while their opposite side acts as repulsive. Reasons for the attractiveness of obstacles are the comfort that can be achieved by leaning against the obstacle. These positions also reduce the number of neighbouring pedestrians especially those standing behind, as well as the feeling of leaving space for passing pedestrians as the space directly next to the obstacle cannot be used for walking. Due to the limited line of sight, the rearward side of obstacles is an unattractive waiting place. Especially narrow obstacles structure the platform visually into two sides and thus guide passengers towards the side where they intend to board the train. The wide obstacle, despite blocking the direct view to the far side of the platform, did not lead to an accumulation of passengers at the side of the entrance, and on the contrary led to a more even distribution of participants along the platform. Hence, despite the intuitive assumption that obstacles narrow the available space, they can be used to guide passengers towards waiting places that are further away from the entrance.

In runs with 40 participants the waiting time was varied. With an increased waiting time some pedestrians were observed to start walking around instead of standing at a fixed position. This behaviour was not observable in runs with a shorter waiting time, indicating that in the case of longer waiting times the desire to optimize the available space becomes more important to certain pedestrians. Over all, an increase of waiting time decreases the comfort of participants. As the runs with 100 participants were only performed with a short waiting time, no clear statement can be made about the influence of waiting time on larger groups of pedestrians. It is, however, expected to decrease the comfort as well. Whether participants still have enough space to walk around while waiting in a larger group is unclear. An increase of waiting time, however, has nearly the same effect on passengers’ evaluation of comfort as a significant increase, here a factor larger than two, of the number of passengers at the platform.

The choice of waiting positions was found to be influenced by different factors, which are identified as staying closer to the entrance rather than walking to the far end of the platform (distance to the entrance), waiting at the side of the next intended action (distance to the exit), keeping a safe distance from the platform’s edges, avoiding places that are often disturbed by newly arriving passengers (flow avoidance) and the repulsive and attractive influence depending on the side of obstacles. A superposition of these factors generates a suitable floor field of attractiveness, that can benefit in the modelling and simulation of waiting passengers at train station platforms. However, this model does not yet take into account that passengers tend to distribute along the platform so that the distances between neighbouring passengers are evenly distributed.

Waiting passengers do not cover the whole available space but favour certain areas. However, inside the crowd the interpersonal distances between neighbouring persons were remarkably similar. Despite the fact that the available space for each person is larger in runs with 40 participants than with 100 participants, the interpersonal distances for all runs are distributed around 1.0–1.2 m. In other words, in the limit of the variations (*N* = 40 and *N* = 100), the distance is independent of the number of passengers on the platform. This leads to the seemingly contradictory statement that the distance between pedestrians is independent of the global density. Whether this finding is valid for higher densities and other geometries as well is an open question. According to the concept of personal space introduced in [36], interpersonal distances between unacquainted pedestrians are expected to be larger than 1.2 m. However, the distance zones in [36] were derived for face-to-face situations while waiting passengers stand aligned behind each other. It is therefore likely that these distance zones need to be adapted depending on the context of the situation. The equal distances determined in this study are consistent with the observations for public spaces in [37]. How this is achieved over large distances (here 20 m) and in complex structures (with obstacles) and which dynamic processes lead to this distribution is unclear. Due to the limited variations in the experiments, no statement can be given whether a longer waiting time would lead to a uniform space usage and coverage of the whole available space.

The results obtained by this experimental study can be transferred to real-life train station platforms under consideration of certain factors. While the experiments were conducted using a two-sided platform where only one train was expected to arrive, in real-life scenarios usually both sides of the track are used for boarding and alighting. Therefore, the waiting phases of passengers waiting for their trains at opposing sides will overlap. The interaction effect between the passengers waiting for trains at different platform sides was not part of this study. Since the length of the experimental platform was much smaller than a real platform, the results cannot be applied to whole platforms but only to certain areas. Due to the simplifications that were necessary in the experiments, at real platforms, the various installations such as information boards, signs indicating the next trains etc. and their repulsive or attractive influences, must to be taken into account. The preferred interpersonal distance of 1.2 m can be used to determine the maximum number of passengers in certain waiting areas which still ensures the passengers’ comfort. Assuming that persons are standing aligned on a grid, interpersonal distances of 1.2 m would lead to Voronoi densities of approximately 0.7 m^−2^. This density value can be an indicator during the planning of waiting areas on platforms.

The results of this study can be used to optimize the pedestrian distribution at railway platforms and thereby increase the robustness of the system during peak loads. The factors influencing pedestrians’ waiting behaviour and distribution are not solely applicable in the context of railway platforms, but can also be derived for other scenarios. It was shown that the key concepts obtained on inflow experiments into small rooms can be extended to reflect the situations at railway platforms. A further expansion will most likely make these concepts suitable for varying fields of applications.

The experiments using a mock-up train platform have shown that even when pedestrians are waiting in such simple spatial structures, complex phenomena can be observed which can only be described by a superposition of several factors. Furthermore, deviations and complex correlations to basic assumptions in pedestrian dynamics are revealed, especially in the relative positioning of passengers between each other. These findings indicate that concepts widely used in pedestrian dynamics, such as the personal space zone described in [36], need to be expanded. When people are aligned, self-organization phenomena lead to equal interpersonal distances which, moreover, are found to be independent of the global density. In particular, it is unclear how pedestrians manage to globally adjust this equality over a distance of 20 m. Since visual signals or globally acting stimuli or instructions can be excluded, it must be a local balancing process which surprisingly leads to a global equal distribution.

## 4. Methods

### 4.1. Experiments

The experiments in this study were conducted from 8 to 10 October 2021 in a multi-purpose hall in Düsseldorf, Germany. On each of the experiment days the participants were divided into three groups and interchanged between three different experimental sides inside the hall periodically. Therewith, three different experiments were performed simultaneously. This article only considers experiments from one side. Details on the other experiments and the overall procedure can be found in [39]. The experiments were conducted during the COVID-19 pandemic and in order to minimize the risk of infection all participants were tested prior to entering the hall and were requested to wear masks at all times. In addition to all safety precautions (rapid test, masks etc.), the participants were getting used to crowded situations by an ‘icebreaker experiment’. They were not informed about this experiment as it was a part of their walking way towards the first experiment side. In the morning, each group was led inside a corridor with two doors on their way to the first experiment. Once all participants were inside, the doors were closed (inside the corridor were densities of about 1 pedestrian m^−2^). After a waiting time of a few minutes the participants were then led to the first experiment. This way the participants experienced dense situations prior to the first experiments. To estimate the extent to which the participant’s behaviour was influenced by the pandemic, they were asked to indicate this in a questionnaire after the last experiment of the day. The questions were answered using a 7-point scale ranging from ‘strongly agree’ (1) to ‘strongly disagree’ (7). Among other questions participants were asked to self-report whether they would have behaved differently before the pandemic. Participants indicated that they did not act differently than they would have before the pandemic (*M* = 2.69) and had already been inside crowds elsewhere since the pandemic had started (*M* = 4.02). A more detailed description on the ice-breaker experiments and the questionnaires regarding pandemic influences can be found in [39]. In total, 1038 participants took part in the experiments, with ages ranging from 18 to 85 years (median: 31 years). In total, 47% were male and 51% female. They were paid for participation. In order to automatically extract participants' head trajectories, the wearing of green caps was mandatory. Each cap was equipped with an individual code, which is assigned to the participant’s data instead of the real name. At the beginning of the experiments, participants were asked how often they use public transportation and 80% of the participants stated they use public transportation on a regular basis at least several times per month. Therefore, participants can be expected to be familiar with the set-up.

### 4.2. Experimental set-up

The experiments were performed by using a mock-up train station platform with a size of 7 × 20 m and a height of 0.8 m as seen in figure 1. Comparable to typical railway platforms in Central Europe, a safety line marked the hazard zone at a distance of 0.8 m from the platform’s edges. The platform was either equipped with no obstacle, with a narrow or a wide obstacle. The obstacles were located in the middle of the platform. The narrow obstacle was 0.6 × 3.6 m in size and the wide obstacle 3 × 3.6 m. Both had a height of 2 m. Obstacle sizes were chosen to represent common platform structures, such as information boards or elevators. The experiment side was separated from other parts of the hall by black curtains so that participants did not see the set-up beforehand. In order to reduce disturbance and to simulate a typical railway environment, a speaker box with a recording of train station sounds was placed below the platform. Participants entered the platform through stairs with a width of 3 m attached to the platform’s smaller side (right-hand side in figure 1). The arrival of a train was simulated by three movable stairs with a width of 1.5 m, which were manoeuvred to their positions for safe attachment at the larger side of the platform (left side in figure 1*d*). The exact position of these stairs was unknown to the participants. Each set-up of the platform, with or without obstacle, was tested with 40 and 100 participants. In runs with 40 participants the waiting time, which started after the last participant of the run had entered the platform, was either 2 or 4 min; in runs with a higher number of participants the waiting time was 2 min. Each participant took part in one run with 40 and one run with 100 participants. Over the course of the 3 days of the experiments, each scenario was repeated three times with different participants, cf. [39].

#### 4.2.1. Choice of experimental conditions

The experimental set-up was designed as a compromise between a realistic configuration and technical feasibility. It was therefore necessary to limit the focus of the study to the factors that were expected to be most relevant and technically possible to investigate. This led to a downsizing of the platform due to the technical effort to cover the complete area by the field of vision of the camera system and the costs of installing technical equipment and the platform itself. The obstacles were chosen as simple as possible but comparable to real-life platform infrastructure. In order to ensure the coverage of the whole experimental platform by the video cameras, the obstacles were placed in the middle of the platform, cf. [47]. The number of participants was planned to be higher, but despite ensuring payment for the participants and publicly advertising the experiments, it was not possible to find more volunteers, which might be caused by the fact that the experiments were performed during the pandemic. Additionally, temporal constrains (e.g. number of experimental days, work load for extraction of trajectories and analysis) and the costs (e.g. technical equipment, payment of staff and participants, platform parts) were limiting the number of possible repetitions of the experimental runs and therewith the number of factors that could be investigated.

### 4.3. Experimental procedure

Before the experiment, participants were guided to a waiting area separated from the experimental set-up by black curtains. Depending on the run (for an overview see table 1), a certain number of participants were given the following instructions (translated from German): ‘Imagine you are at a train station. Behind those curtains is the platform, which you will enter through the stairs. You plan to take the train that will arrive in a few minutes at the platform at the left-hand side.’ After the instructions, the participants’ inflow to the platform was regulated so that approximately one person entered the platform every 3–5 s. Participants then waited for a predefined waiting time (either 2 or 4 min; see table 1). After the waiting time was completed, the train arrival was announced, the movable stairs were positioned and the participants left the experimental side and were guided in a different waiting area. In this second waiting area, a mood button terminal was placed, which participants were asked to use after each run. A mood button terminal is a tablet with four smiley buttons, which people passing by can use to express a feeling by pressing one of the buttons. The question displayed on the terminal was ‘How did you feel during this experiment?’ (translated from German). The terminal saved a timestamp and the pressed mood button, which had to be chosen from ‘very happy’, ‘happy’, ‘unhappy’ and ‘very unhappy’. In order to ensure that participants used the terminals, they were actively reminded to do so after each run. In runs in which questionnaires were to be filled out (as indicated in table 1), those were distributed in the second waiting area. Questionnaires were either distributed for both runs with 40 participants (for the set-ups with narrow or wide obstacle) or for the runs with 100 participants (for the set-up without obstacle). Participants were asked how they rated the following items (translated from German): (i) I perceived the space available for me as sufficient. (ii) I perceived the density at the platform as unpleasant. The rating was a 7-point scale ranging from ‘1 = strongly disagree’ to ‘7 = strongly agree’.

### 4.4. Data collection and preparation

The trajectory data were collected by filming the experimental set-up with three cameras facing straight down. Two of these cameras were used to cover the platform with an overlap of camera views in the middle; the third camera filmed the entrance staircase and was used to read the individual code markers on the participants’ heads. A detailed description on camera configuration and techniques can be found in [39]. Participants’ head trajectories were automatically extracted following [40] using the software PeTrack [41], which is achieved by recognizing and tracking of the green caps. Trajectories of all runs were then manually corrected and the different camera views were combined to result in one complete trajectory set for each run. The resulting trajectories consist of an unique ID number (the number of the marker on the participants’ cap) and the x- and y-positions at a given time frame. In this study, the frame rate is 50 frames per second. The obtained trajectories were then used for the analysis outlined in the next section.

## Data Availability

All raw data, i.e. video recordings and head trajectories, are available through the Pedestrian Dynamics Data Archive hosted by Forschungszentrum Jülich and can be found here: http://ped.fz-juelich.de/da/2021train_platform [48].
